# Procalcitonin as a biomarker for severe *Plasmodium falciparum *disease: a critical appraisal of a semi-quantitative point-of-care test in a cohort of travellers with imported malaria

**DOI:** 10.1186/1475-2875-8-206

**Published:** 2009-09-01

**Authors:** Dennis A Hesselink, Jan-Steven Burgerhart, Hanna Bosmans-Timmerarends, Pieter Petit, Perry JJ van Genderen

**Affiliations:** 1 Department of Internal Medicine, Erasmus MC, Rotterdam, the Netherlands; 2 Department of Internal Medicine, Harbour Hospital and Institute for Tropical Diseases, Haringvliet 2, 3011 TD Rotterdam, the Netherlands; 3 Department of Microbiology, Vlietland Hospital, Schiedam, the Netherlands

## Abstract

**Background:**

Imported malaria occurs as a relatively rare event in developed countries. As a consequence, most clinicians have little experience in making clinical assessments of disease severity and decisions regarding the need for parenteral therapy or high-level monitoring. In this study, the diagnostic accuracy of procalcitonin (PCT) for severe *Plasmodium falciparum *disease was assessed in a cohort of 100 consecutive travellers with various species of imported malaria.

**Methods and results:**

In all patients, PCT was measured on admission with a semi-quantitative 'point-of-care' test. Patients with severe *P. falciparum *malaria had significantly higher median PCT levels on admission as compared with patients with uncomplicated *P. falciparum *disease. In addition, PCT levels in patients with non-*falciparum *malaria were also higher compared with patients with non-severe *falciparum *malaria but lower compared with severe *P. falciparum *malaria. At a cut-off point of 10 ng/mL, PCT had a sensitivity of 0,67 and a specificity of 0,94 for severe *falciparum *disease. However, at lower cut-off points the specificity and positive predictive value were rather poor although the sensitivity and negative predictive value remained high.

**Discussion:**

Potential drawbacks in the interpretation of elevated PCT levels on admission may be caused by infections with non-*falciparum *species and by concomitant bacterial infections.

**Conclusion:**

Semi-quantitative determination of PCT on admission is of limited use in the initial clinical assessment of disease severity in travellers with imported malaria, especially in settings with limited experience with the treatment of malaria.

## Background

Imported malaria occurs as a relatively rare event in developed countries. As a consequence, most clinicians have little experience in making clinical assessments of disease severity and decisions regarding the need for parenteral therapy or high-level monitoring. In addition, obtaining a correct diagnosis of malaria may be troublesome in centres where laboratory staff are less skilled in preparing malaria thick and thin smears and in the proper identification of the causative *Plasmodium *species and quantification of the parasite load [1]. These centres usually rely on rapid diagnostic tests for the diagnosis of malaria. However, these tests do not provide any information about the severity of the infection, which is mandatory for evaluation of *Plasmodium falciparum *infections. Therefore, well-validated, simple, laboratory-based prognostic markers would be of great value in assisting clinicians dealing with returned travellers with malaria. Unfortunately, many laboratory markers including parasitaemia have poor sensitivity, specificity and predictive values for estimating the severity of disease [2].

Procalcitonin (PCT), a precursor peptide from the hormone calcitonin, has been considered by some, but not all, to be a specific and useful indicator of systemic bacterial infections [3,4]. Interestingly, elevated PCT levels (measured with quantitative assays), were also demonstrated in several patient series with malaria [5-11]. In the present study, the diagnostic accuracy of PCT for severe *P. falciparum *disease was evaluated in a cohort of 100 consecutive travellers with various imported malaria species. For this purpose, the PCT-Q^® ^test was used, a semi-quantitative immunochromatographic "point-of-care" test, which, in contrast to quantitative PCT assays, allows a rapid measurement of PCT without a need for further technical equipment or support and can be easily incorporated in the initial clinical assessment of travellers who return home ill.

## Methods

### Patient selection

The Harbour Hospital is a 161-bed general hospital located in Rotterdam, the Netherlands. It also harbours the Institute of Tropical Diseases, which serves as a national referral centre for tropical diseases. All patients reported herein were consecutive travellers diagnosed with imported malaria, who were admitted to the Institute of Tropical Diseases of the Harbour Hospital in the period January 2004 to December 2007 and participated in the PCTTM (ProCalciTonin in Travel Medicine) observational survey. The PCTTM was an observational study that had the aim of evaluating the diagnostic accuracy of the semi-quantitative PCT-Q^® ^test (see below) in the initial assessment of travellers who return home sick after a stay in the tropics or sub-tropics. Of each traveller demographic, clinical and laboratory data were collected including a blood sample on admission for PCT testing. Patients were classified as having severe or complicated *P. falciparum *malaria if they met pre-specified criteria for severe malaria [2,12].

### Laboratory methods

Routine examinations included red blood cell count, haematocrit, white blood cell count, platelet count, serum electrolytes, total bilirubin, serum creatinine, liver enzymes, blood glucose and plasma lactate. Blood smears (thin and thick films), were obtained from finger pricks and stained with Giemsa and parasite counts were performed. Malaria was diagnosed by QBC analysis, *PfHRP*-2 screening and conventional microscopy with subsequent specification of the *Plasmodium *species (*Plasmodium falciparum*, *Plasmodium vivax*, *Plasmodium ovale *or *Plasmodium malariae*, respectively). In case of *P. falciparum *infections, parasite density was expressed as the number of *P. falciparum *trophozoites per 100 red cells in a thin film or the number of parasites per 100 white blood cells in a thick film. The parasite load was calculated from these figures using the actual number of white or red blood cells counted in the blood sample.

PCT was measured with the BRAHMS PCT-Q^® ^test (Brahms Diagnostics, Germany) according to the instructions of the manufacturer. PCT results were classified as follows: a negative result or a PCT < 0,5 ng/ml was classified as "*normal*"; a PCT of 0,5 ng/ml or a PCT between 0,5-2,0 ng/ml was classified as "*low*"; a PCT of 2,0 ng/ml or a PCT between 2,0-10,0 was classified as "*moderate*"; a PCT of 10,0 ng/ml and above 10 ng/ml was classified as "*high*" as recommended [13].

### Statistical methods

All values are presented as median (range). For comparison between groups, the Mann-Whitney U test, Kruskal-Wallis test or Chi square test were used as appropriate. Post-hoc analysis was performed using Mann-Whitney U test. P values at α < 0.05 were considered statistically significant.

## Results

One hundred patients with imported malaria were included in this study. Twenty-nine travellers acquired a non-*P. falciparum *malaria infection (*P. vivax *n = 23; *P. ovale *n = 4; *P. malariae *n = 2), 65 travellers had an uncomplicated *P. falciparum *infection, whereas another six patients had severe *P. falciparum *disease. Thirty-one patients with uncomplicated *P. falciparum *disease, one patient with severe *P. falciparum *disease and eight patients with non-*P. falciparum *disease were considered to be semi-immune or partially immune, respectively. As depicted in Table 1, patients with a severe *P. falciparum *infection had the highest serum CRP, lactate, creatinine, lactate dehydrogenase and bilirubin concentrations and the lowest haemoglobin concentrations and platelet counts on admission. As shown in Table 2, the median PCT concentrations differed significantly between the three groups (Chi-square 17.8; P < 0.001). The lowest median PCT concentration was observed in the group of patients with non-severe *P. falciparum *infections, whereas patients with severe *P. falciparum *infections had the highest median PCT concentration (Table 2).

**Table 1:** General characteristics and laboratory findings on admission of 100 consecutive travellers with imported malaria, subdivided by causative *Plasmodium *species and disease severity.

	Non-*P. falciparum *malaria	Uncomplicated *P. falciparum *malaria	Severe *P. falciparum *malaria	P value
	(n = 29)	(n = 65)	(n = 6)	
Male/female	22/7	47/18	4/2	0,8790
Age (year)	36 (15 - 62)	36 (9 - 67)	48 (29 - 55)	0,2077
Continent of acquisition				
*Africa*	11	62	5	
*Asia*	11	2	-	
*South America*	6	-	1	
Parasite load (trophozoites per uL)	-	3422 (23 - 385000)	174900 (80500 - 567000)	0,0002
Plasma lactate (mmol/L)	1,3 (0,7 - 2,2) *	1,5 (0,9 - 4,4) *	2,7 (1,8 - 5,8)	0,0083
ESR (mm/h)	19 (3 - 82)	16 (1 - 73)	17 (11 - 54)	0,1400
Haemoglobin (mmol/L)	8,0 (4,6 - 10,1)	8,7 (5,2 - 11,1) °	7,4 (4,0 - 10,2)	0,0297
Leucocytes (× 10^9^/L)	4,7 (1,9 - 9,3)	4,9 (1,8 - 9,0)	6,6 (3,2 - 18,5)	0,3867
Platelets (× 10^9^/L)	95 (28 - 167) *	80 (11 - 247) *	22 (3 - 36)	0,0010
C-reactive protein (mg/L)	84 (18 -208) *	109 (5 - 280) ^#^	175 (151 - 265)	0,0056
Plasma glucose (mmol/L)	6,6 (4,9 - 8,2)	6,8 (4,1 - 14,9)	5,9 (4,5 - 7,6)	0,3100
Serum creatinine (umol/L)	91 (62 - 223) *	95 (56 - 208) *	153 (112 - 199)	0,0028
Serum LDH (U/L)	474 (278 - 1133) *	554 (238 - 1234) *	1272 (787 - 2216)	0,0011
Serum total bilirubin (umol/L)	24 (10 - 84) *	26 (4 - 164) *	65 (39 - 269)	0,0033

* P < 0.01, compared with severe *P. falciparum *malaria

° P < 0.05, compared with non-*P. falciparum *malaria

^# ^P < 0.05, compared with severe *P. falciparum *malaria

**Table 2:** Procalcitonin (PCT) levels on admission in relation to causative *Plasmodium *species.

PCT	Non-*P. falciparum *malaria	Uncomplicated *P. falciparum *malaria	Severe *P. falciparum *malaria	
Normal	4	23	0	27
Low	5	16	0	21
Moderate	14	22	2	38
High	6	4	4	14
	29	65	6	100

Non-*P. falciparum vs *uncomplicated *P. falciparum *p = 0.004

Non-*P. falciparum vs *severe *P. falciparum *p = 0.031

Uncomplicated *P. falciparum vs *severe *P. falciparum *p < 0.001

**Figure 1:**
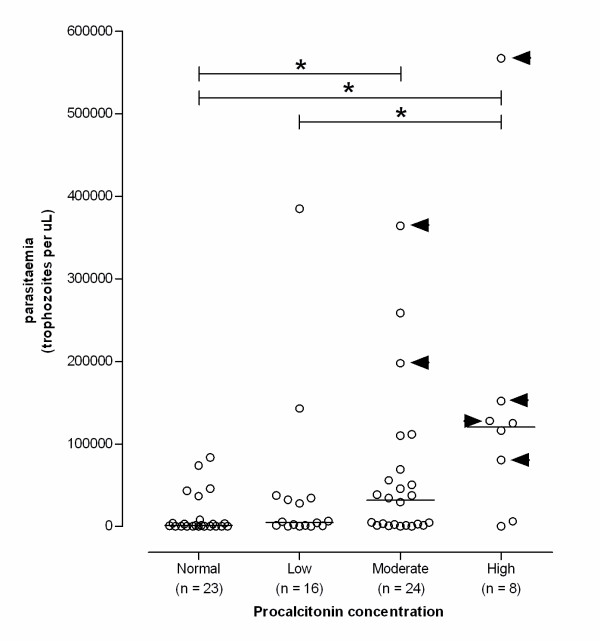
**Procalcitonin (PCT) levels on admission in relation to parasite load in 71 patients with *falciparum *malaria**. Bars represent the median value of each group. Arrow heads indicate the patients with severe *falciparum *malaria. Statistical analysis showed an overall statistically significant difference between the various PCT groups on admission (p = 0.0012). Post-hoc group-to-group analysis showed significant differences in parasite load (identified by the asterix sign *) between various PCT groups (Normal *vs *moderate, p = 0.0044; normal *vs *high, p = 0.0013; and low *vs *high, p = 0.0403, respectively).

When the groups of patients with *P. falciparum *infections were combined and analysed together, a significant correlation between parasitaemia and PCT concentrations was observed. Patients with a high PCT concentration had a significantly higher parasitaemia compared with patients who had a low or moderate PCT concentration (Figure 1). Interestingly, in all patients with severe *P. falciparum *malaria the elevated PCT levels on admission were classified as moderate (n = 2) or high (n = 4), respectively. Depending on the selected cut-off point (Table 3), PCT had an excellent sensitivity for severe *P. falciparum *disease, whereas specificity was poor. At a cut-off point of 10 ng/mL, the test characteristics became clinically relevant with a sensitivity of 0,67 and a specificity of 0,94 for severe *P. falciparum *disease.

**Table 3:** Descriptive statistics of the accuracy of procalcitonin (PCT) as a diagnostic tool for severe *Plasmodium falciparum *disease using various cut-off points.

	PCT cut-off point		
*P. falciparum species only *(n = 71)	*0,5 ng/ml*	*2,0 ng/mL*	*10,0 ng/mL*
Sensitivity	1,00 [n.a.]	1,00 [n.a.]	0,67 [0,56-0,78]
Specificity	0,35 [0,24-0,46]	0,60 [0,49-0,71]	0,94 [0,88-1,00]
Positive predictive value	0,13 [0,05-0,21]	0,19 [0,10-0,28]	0,50 [0,38-0,62]
Negative predictive value	1,00 [n.a.]	1,00 [n.a.]	0,97 [0,93-1,00]
			
*All plasmodium species *(n = 100)	***0,5 ng/ml***	***2,0 ng/mL***	***10,0 ng/mL***
Sensitivity	1,00 [n.a.]	1,00 [n.a.]	0,67 [0,58-0,76]
Specificity	0,29 [0,20-0,38]	0,51 [0,41-0,61]	0,94 [0,83-0,95]
Positive predictive value	0,08 [0,03-0,13]	0,19 [0,06-0,18]	0,50 [0,20-0,38]
Negative predictive value	1,00 [n.a.]	1,00 [n.a.]	0,97 [0,95-1,00]

Data is shown as proportion [95% confidence interval].

## Discussion

In this study, a semi-quantitative PCT test was evaluated as a diagnostic tool for severe *P. falciparum *disease because this 'point-of-care' test may be easily incorporated in the initial clinical assessment of travellers who return home ill, without a need for further technical equipment or support. To that end, PCT levels on admission were studied in a broad spectrum of manifestations of imported malaria, ranging from uncomplicated to severe *P. falciparum *malaria, as well as in non-*falciparum *malaria. Interestingly, in all patients with severe *P. falciparum *disease a moderate to high level of PCT was seen on admission, which agrees with observations made by others [6,8]. Some authors even suggested that an elevated PCT has prognostic value based on observations that PCT levels > 25 ng/mL were related to a fatal outcome in six of seven patients with severe *P. falciparum *disease [6]. However, these assumptions were not validated in some other studies where PCT levels on admission were even higher (up to 662 ng/mL) but without associated case-fatalities [8].

Interestingly, although severe or fatal malaria rarely results from infections with the non-sequestering *Plasmodium *species, *P. vivax*, *P. ovale *and *P. malariae*, increased PCT levels were observed in the majority of these patients on admission as well. Although speculative, these findings may imply that the mechanism whereby PCT levels increase in severe *P. falciparum *disease do not accurately reflect pivotal pathophysiological events in complicated *P. falciparum *disease, like sequestration of infected red blood cells in the microcirculation of vital organs. Rather, the elevation of PCT may be the result of activation of a common host response to malaria parasites. In fact, some even suggest that *P. falciparum *malaria *per se *is not associated with a stronger host response than *P. vivax *or *P. ovale *malaria, but that the density of the infection of the causative *Plasmodium *species influences the extent of the host response [14].

In the present study, PCT had an acceptable to excellent sensitivity for severe *P. falciparum *disease and a high negative predictive value. Given the low percentage of false negatives (*i.e*. patients with a low PCT on admission who nonetheless experienced a complicated course of malaria), the use of semi-quantitative PCT levels on admission to guide to treatment, would result in only very few patients with severe *P. falciparum *malaria being denied high-level monitoring and intensive treatment. However, the specificity and positive predictive value of the semi-quantive PCT test were rather poor. Applying semi-quantitative PCT levels on admission as a guide to therapy would therefore lead to a large proportion of patients receiving more intensive monitoring and treatment than strictly necessary. In our opinion, only at a PCT cut-off point of 10 ng/mL for severe *P. falciparum *disease, the test characteristics were clinically valuable. Moreover, in settings were the prevalence of malaria is lower than that reported herein, the positive predictive value of PCT will probably be even lower resulting in more false positive tests.

Possibly, the overall sensitivity of PCT for the prediction of the course of malaria, could be improved if PCT was measured more precisely by use of an ultrasensitive PCT assay instead of the semi-quantitative assay used in this study. Several studies have directly compared the performance of both techniques and demonstrated satisfactory but not complete, concordance [13,15]. However, in the latter study [15], a satisfactory concordance was only reached when results in the next category were included to account for readout errors, which may further compromise the applicability of this semi-quantitative test in clinical practice.

Another drawback on the use of PCT for estimating malaria severity might be the occurrence of bacterial co-infection, which may also lead to elevated PCT levels on admission. In a large French intensive care unit study on imported severe *falciparum *malaria, 13 of 93 (14%) patients with severe imported malaria were diagnosed with a concomitant bacterial infection, which contributed to a poor outcome [16]. However, in this study, blood cultures were routinely taken on admission in case of fever and remained negative, making the occurrence of concomitant bacteraemia a less likely event.

## Conclusion

Severe *P. falciparum *disease and non-falciparum malaria are associated with elevated PCT levels on admission. Although determination of PCT may be of some help in the initial clinical assessment of disease severity in travellers with falciparum malaria in hospitals with extensive experience with the treatment of malaria, the overall test characteristics were too poor to advocate a general use of this semi-quantitative PCT assay. Potential drawbacks in the interpretation of elevated PCT levels on admission may be caused by infections with non-*falciparum Plasmodium *species and by concomitant bacterial infections.

## Competing interests

The authors declare that they have no competing interests.

## Authors' contributions

DAH participated in the data collection, performed the statistical analysis and data interpretation and contributed to the writing of the manuscript. JSB and HBT participated in the data collection and contributed to the writing of the manuscript. PP performed the procalcitonin assays, undertook the data collection and contributed to the manuscript. PVG conceived and designed the study, carried out the main analysis and wrote the manuscript. All authors approved the final version of the manuscript.
